# Short Read Lengths Recover Ecological Patterns in 16S rRNA Gene Amplicon Data

**DOI:** 10.1111/1755-0998.14102

**Published:** 2025-03-13

**Authors:** Stephanie D. Jurburg

**Affiliations:** ^1^ Department of Environmental Microbiology Helmholtz Centre for Environmental Research—UFZ Leipzig Germany; ^2^ German Centre for Integrative Biodiversity Research (iDiv) Halle‐Jena‐Leipzig Leipzig Germany

**Keywords:** bacteria, bioinformatics, data reuse, metabarcoding, microbiome

## Abstract

16S rRNA gene metabarcoding, the study of amplicon sequences of the 16S rRNA gene from mixed environmental samples, is an increasingly popular and accessible method for assessing bacterial communities across a wide range of environments. As metabarcoding sequence data archives continue to grow, data reuse will likely become an important source of novel insights into the ecology of microbes. While recent work has demonstrated the benefits of longer read lengths for the study of microbial communities from 16S rRNA gene segments, no studies have explored the use of shorter (< 200 bp) read lengths in the context of data reuse. Nevertheless, this information is essential to improve the reuse and comparability of metabarcoding data across existing datasets. This study reanalyzed nine 16S rRNA datasets targeting aquatic, animal‐associated and soil microbiomes, and evaluated how processing the sequence data across a range of read lengths affected the resulting taxonomic assignments, biodiversity metrics and differential (i.e., before‐after treatment) analyses. Short read lengths successfully recovered ecological patterns and allowed for the use of more sequences. Limited increases in resolution were observed beyond 150 bp reads across environments. Furthermore, abundance‐weighted diversity metrics (e.g., Inverse Simpson index, Morisita‐Horn dissimilarities or weighted Unifrac distances) were more robust to variation in read lengths. Read lengths alone contributed to consistent increases in the total number of ASVs detected, highlighting the need to consider metabarcoding‐derived diversity estimates within the context of the bioinformatics parameters selected. This study provides evidence‐based guidelines for the processing of short reads.

## 1 Introduction

The 16S rRNA gene is approximately 1550 bp long and encodes the small subunit ribosomal RNA molecules of ribosomes. Originally used by Woese and Fox to examine the phylogeny of prokaryotes (Woese and Fox 1977), the 16S rRNA gene serves as a molecular clock (Woese 1987), as a means for differentiating prokaryotic taxa, and to evaluate microbial diversity (Giovannoni et al. 1990; Stackebrandt and Goebel 1994). Sequencing 16S rRNA gene amplicon sequences from mixed samples (i.e., metabarcoding) has revolutionised microbial ecology, especially with the advent of high throughput sequencing. Metabarcoding of the 16S rRNA gene has helped to identify the ‘unculturable majority’ of bacterial taxa (Lloyd et al. 2018) and revealed the astounding complexity and ubiquity of microbes globally (Thompson et al. 2017).

The structure of the 16S rRNA transcript and its essential function in protein synthesis have limited the rate of evolutionary change in the gene, resulting in highly conserved regions that can be leveraged as primer targets that flank variable regions for sequencing (Clarridge 2004). Initial sequence‐based assessments of prokaryotic diversity relied on cloning and Sanger sequencing, which could sequence the entirety of the 16S rRNA gene from a few reads at a high cost and effort. The advent of next‐generation sequencing technologies (e.g., Illumina, 454 pyrosequencing, Ion Torrent), hereafter amplicon sequencing, allowed for the sequencing of a much greater number of sequences, but at a length of < 600 base pairs (Caporaso et al. 2011), resulting in sequences of part of the target marker gene, or partial metabarcodes.

Despite the development of novel techniques that allow the sequencing of the full length of 16S rRNA genes (e.g., Nanopore or SMRT sequencing) and provide a higher taxonomic resolution (Johnson et al. 2019; Matsuo et al. 2021), amplicon sequencing of shorter segments (i.e., partial metabarcodes) remains the most accessible method for the identification of microbial communities. Amplicon sequencing data continue to grow exponentially in sequence archives (Jurburg et al. 2020), representing an important data resource for future research, and have already provided important insights into the diversity of prokaryotes (e.g., Louca et al. 2019). Each step of the sequencing data production process, from the sampling design in situ through choices made during amplification (Brooks et al. 2015; Schloss et al. 2011) and bioinformatics processing (Kang et al. 2021; Marizzoni et al. 2020; Prodan et al. 2020) affects the resulting microbial diversity estimates (Zinger et al. 2019) and greatly complicates the reuse of metabarcoding data in a synthetic context, where different studies often apply slight variations in their experimental and laboratory techniques.

Choices made from the sample collection through the sequencing steps cannot be modified; however, sequence data are generally archived in their raw format, providing an opportunity to reduce differences between datasets during bioinformatics processing. Despite early work highlighting the importance of read numbers over read lengths in capturing ecological patterns in microbial communities (Liu et al. 2007) and the importance of applying consistent sequencing approaches (e.g., primer region) to improve the comparability of metabarcoding data (Wasimuddin et al. 2020), no studies have explored the effect of using shorter read lengths as a tool for improving the comparability of reused sequence data.

The selection of a target segment from the sequenced region (i.e., truncation) is a common practice that removes lower‐quality read ends (Callahan, McMurdie, et al. 2016), but can also serve to select the same target region across metabarcoding datasets, reducing technical biases between them. While full 16S rRNA gene sequences inherently contain greater taxonomic information and produce taxonomic assignments of higher quality (Curry et al. 2022; Johnson et al. 2019), the extent to which short read lengths (i.e., < 200 base pairs) are able to recover higher‐level taxonomic assignments and ecological patterns has received little attention. Understanding the opportunities and limitations of shorter 16S rRNA gene read lengths is essential, especially for the reuse of rapidly growing sequence data archives (Jurburg et al. 2020). Existing bioinformatics workflows recommend maximising read length while preserving high‐quality segments (e.g., (Callahan, Sankaran, et al. 2016)); however, allowing shorter read lengths can improve the comparability of sequences across datasets, allowing for re‐analyses that target the identical 16S rRNA gene region and avoid biases that emerge from sequencing different, but overlapping target regions (Bukin et al. 2019; Tremblay et al. 2015; Yang et al. 2016; Yu et al. 2008) or from differential read lengths. Characterising the impact of shorter read lengths on 16S rRNA gene‐based ecological assessments may also serve for the integration of data from diverse platforms that produce a range of sequence lengths (e.g., from full gene sequencing with Nanopore to 150 bp with single‐ended sequencing in Illumina HiSeq).

To examine the effect of sequence length on microbial diversity estimates, nine datasets from disturbed soil, water, and animal microbiomes sequenced using the same primer set and sequencing platform across a gradient of read lengths were reprocessed. It was hypothesised that (1) shorter reads would result in an increase in the number of unclassified ASVs at finer taxonomic resolutions and (2) lower diversity estimates, but that (3) the signal of the response of bacterial communities to disturbance would still be detectable as changes in the taxonomic or phylogenetic composition of the community resulting from the disturbance.

## 2 Materials and Methods

### 2.1 Reused Data

To examine the effect of read length on diversity estimates, publicly available data from nine experimental datasets targeting the soil (Fuentes et al. 2016; Jurburg et al. 2017; van Kruistum et al. 2018), water (Dong et al. 2017; Qian et al. 2017) and animal (Jurburg et al. 2019; Kennedy et al. 2018; Venkataraman et al. 2016) microbiomes before and immediately after (i.e., 0–2 days) a single, strong disturbance were selected (Table 1). With the exception of datasets 2 and 4, which had experimental triplicates (Fuentes et al. 2016), all datasets had at least quadruplicate samples per time point and per condition (i.e., pre‐ and post‐disturbance). When a study contained more than four replicates, the five largest files for each *dataset* × *time* combination were selected. A list of the datasets used, their accession numbers, and their file sizes is available in Table S1. All datasets targeted the 515‐806 (i.e., primarily the V4) region of the 16S rRNA gene. This region was selected due to its wide use among the scientific community and the availability of standardised protocols through the Earth Microbiome Project (Thompson et al. 2017), its sensitivity to a broad range of bacterial diversity (Zhang et al. 2023), and its common use in microbiome studies, which has resulted in increased availability of data targeting this region. Further information on the data's origin and accessibility, an experimental description, the disturbance applied, the DNA extraction method employed, and the times of sampling are available in Table S1.

**TABLE 1:** Description of datasets used in this study. Further details about each study and the specific samples used are available in Table S1.

ID	Title	Environment	Disturbance	Accession	Rarefaction	Avg file size (Mb)	Original reads
1	Successional dynamics in the gut microbiome determine the success of *Clostridium difficile* infection in adult pig models	Pig faeces	Clindamycin added	PRJNA528235	22,946	10.3	66,357
2	Temporal dynamics of gut microbiota in triclocarban‐exposed weaned rats	Rat faeces	TCC added	PRJNA351840	43,594	11.7	134,866
3	Variable responses of human microbiomes to dietary supplementation with resistant starch	Human faeces	Butyrate diet	PRJNA306884	42,448	6.5	55,000
4	Temporal dynamics of bacterioplankton communities in response to excessive nitrate loading in oligotrophic coastal water	Seawater (35 L)	Nitrate added	PRJDB5020	28,368	4.8	66,745
5	Weathered crude oil alters biogeochemical cycles and microbial populations in permeable subtidal sediments	Seawater (8.5 L)	Oil added	NA	23,869	6.9	42,436
6	Alteration in successional trajectories of bacterioplankton communities in response to co‐exposure of cadmium and phenanthrene in coastal water microcosms	Seawater (42 L)	Cadmium added	PRJDB4992	29,435	3.9	47,517
7	From rare to dominant: a fine‐tuned soil bacterial bloom during petroleum hydrocarbon bioremediation	Soil (300 g)	Diesel added	PRJNA291526	400	4.4	24,295
8	Differential responses of RNA and DNA during secondary succession in soil microbiomes	Soil (50 g)	Heat shock	PRJNA329541	43,025	20.5	99,260
9	Resistance and Recovery of Methane‐Oxidising Communities Depends on Stress Regime and History; A Microcosm Study	Soil (Petri dish)	Ammonium added	PRJEB24893	24,434	6.8	52,001

### 2.2 Sequence Processing and Data Analysis

Sequence data and metadata were downloaded from NCBI (Table S1). All processing was done with the *dada2* pipeline (Callahan, McMurdie, et al. 2016) using only the forward reads. Importantly for sequence data reuse, reverse reads are often not available in archived sequence data (Jurburg et al. 2020), either because pair‐ended sequencing was not performed or the reverse reads are not archived. In the case of datasets 6 and 7, paired reads were merged prior to archiving. For each sample, read length was reduced from 200 to 60 bp in intervals of 10 bp. Reads were truncated by varying the *truncLen* parameter of the *FilterAndTrim* function of *dada2* (i.e., truncating a read 60 bp from the 5′ end), with all other parameters kept at the same standard values (maxN = 0; maxEE = 2, truncQ = 2). This range of read lengths was selected as it represents the minimum output of all modern sequencing technologies, including HiSeq. In this study, datasets had an average read length of 150 bp. Taxonomy was assigned using SILVA v138 (Quast et al. 2012) using the dada2 algorithm, and phylogenetic trees were created using the *phangorn* package (Schliep 2011), according to standard protocols (Callahan, Sankaran, et al. 2016). For all samples, the number of unassigned reads at each taxonomic level, and the percentage of original reads included in the final ASV table was recorded.

ASV tables were analysed in R version 4.1.0 (R Core Team 2014) using the *phyloseq* (McMurdie and Holmes 2013) and *vegan* (Oksanen et al. 2007) packages. To compare diversity estimates, all versions of each dataset were rarefied to the lowest number of reads (see Table 1). Given that the accuracy of chimera checking algorithms decreases with decreasing read length and is not recommended for reads shorter than 100 bp (Wright et al. 2012), no chimera checking was performed. To explore the impact of read length on the quantification of microbial alpha diversity, the pre‐disturbance samples of each dataset were selected to measure richness and inverse Simpson diversity (i.e., Hill numbers; *q* = 0 and *q* = 2 respectively; (Chao et al. 2014)), which are more heavily weighted by the rare and dominant taxa, respectively, as well as Faith's phylogenetic diversity (Faith 2002), which considers the phylogenetic tree resulting from shorter read lengths. Calculations were performed using the *HillR* (Li 2018) and *picante* (Kembel et al. 2010) packages. Similarly, to explore the effects of read length on beta diversity within the Hill framework, Sorensen (*q* = 0) and Morisita‐Horn (*q* = 2) dissimilarities were calculated using the *vegan* package. Unifrac distances (weighted and unweighted) were also calculated using the *phyloseq* package, as they consider phylogenetic diversity. To assess the extent to which read length affected the ecological conclusions derived from the data, samples from before and shortly after disturbance for each dataset were compared. For alpha diversity, undisturbed and disturbed samples were compared using nonparametric Wilcoxon tests, and for beta diversity, undisturbed and disturbed samples were compared using a PERMANOVA (*adonis2*) for each read length. Finally, to examine the loss of resolution with decreasing read length, a mantel test of the beta diversity metrics between the longest read length (200 bp) and all shorter reads was performed for each dataset. To assess whether read lengths affected these metrics consistently, nonparametric Mann–Kendall tests were performed.

## 3 Results

The sequence data recovered from INSDC databases had an average read length of between 150 (study 2) and 600 bp (study 6), and a wide range of sequence qualities (Figure S1), which indicated that two of the datasets (studies 6 and 7) were merged prior to archiving. With the exception of samples from dataset 7, read qualities remained high up to 200 bp (Figure S1). Prior to processing, the average number of reads per sample varied widely across datasets from 24,295 reads (dataset 7) to 134,866 in dataset 2 (Table 1).

**FIGURE 1:**
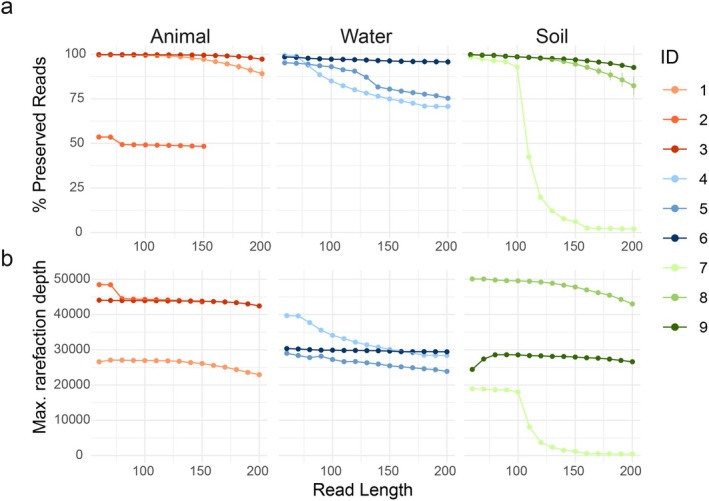
The effect of read length on the number of reads preserved after processing (a) and rarefaction (b). In a, points represent means for all samples from a dataset reprocessed in this study, and error bars represent the standard deviation. In (b) the maximum rarefaction depth was calculated as the minimum number of reads in a study for a given read length.

Processing sequence data across a gradient of read lengths had consistent effects across datasets (Figure 1). With longer reads, a lower percentage of reads was conserved across all datasets after processing (Kendall's tau −0.94 to −0.45; *p*‐value < 0.001 for all comparisons), with a decreasing percentage of the original reads being preserved with the average number of reads conserved per sample decreasing gradually with read length in data (Figure S2). Consequently, the maximum possible rarefaction depth decreased with longer reads (Kendall's tau −1 to −0.52; *p*‐value < 0.007 for all comparisons).

**FIGURE 2:**
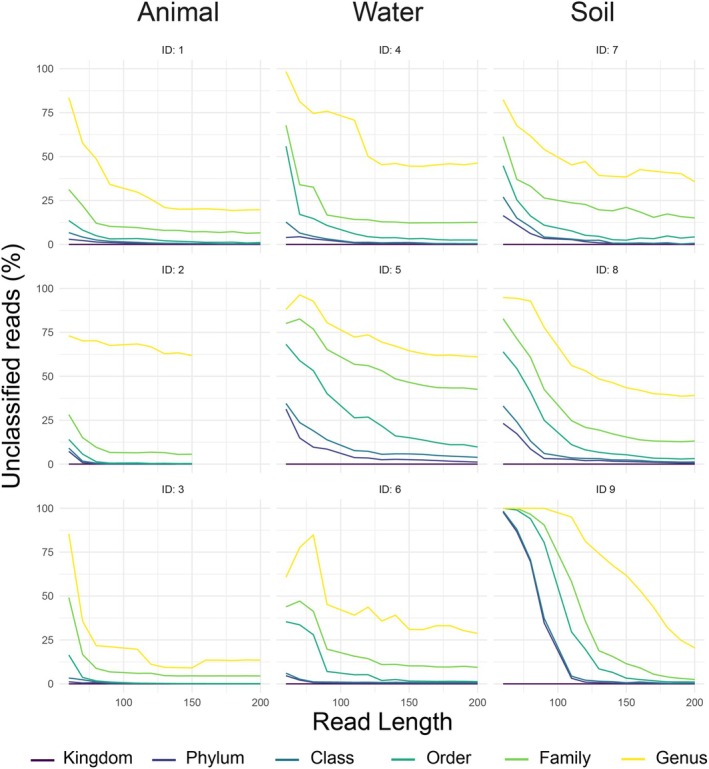
Relationship between taxonomic classification and read length for the undisturbed samples in each dataset. For each taxonomic level, unclassified reads indicate the average percentage of reads in the study sample set that did not receive a taxonomic assignment.

The percentage of reads without taxonomic classification decreased sharply with read length (Figure 2). With the exception of dataset 2, the percentage of unclassified reads across all taxonomic levels decreased with longer read lengths (Kendall's tau < 0.45, *p*‐value < 0.05 for all comparisons). As expected, this pattern was most pronounced at the finer taxonomic levels, but depended on the diversity and/or prior characterisation of the system. In the better characterised animal microbiomes, longer reads resulted in only marginal improvements to classification. 150 bp reads were sufficiently long to result in the genus‐level classification of 70%, 53%, and 52% of the reads in animal, aquatic and soil microbiomes, respectively.

**FIGURE 3:**
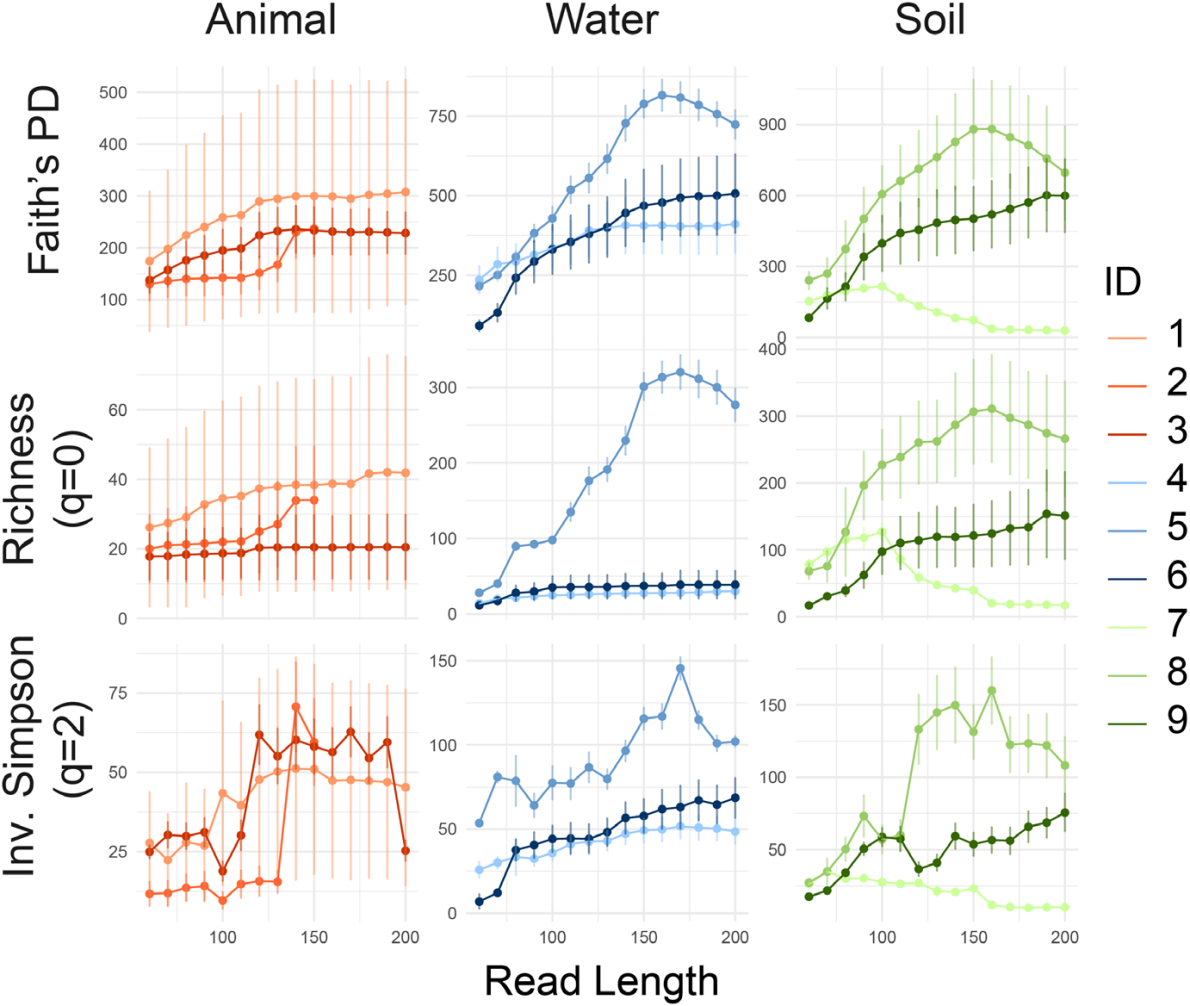
Relationship between alpha diversity and read length, calculated as Faith's phylogenetic diversity, richness (*q* = 0) and inverse Simpson's index (*q* = 2). Points and error bars represent means and error bars for undisturbed samples in each dataset, for each read length. The result of Wilcoxon tests between the alpha diversity in disturbed and undisturbed samples is presented in Figure S2.

To determine how trimming affected the estimation of diversity, the richness, inverse Simpson index, and Faith's phylogenetic diversity were calculated for the undisturbed samples in each dataset. With the exception of dataset 7, diversity increased consistently with read length. Richness increased significantly (Kendall's tau 0.52–0.99; *p*‐value < 0.007 for all comparisons), but saturated more rapidly in less diverse environments (i.e., animal microbiomes; Figure 3). Inverse Simpson's index exhibited a stronger pattern (Kendall's tau 0.69–0.99; *p*‐value < 0.001 for all comparisons), while Faith's PD exhibited a weaker one, with datasets 2 and 3 exhibiting no significant relationship. For all metrics, datasets achieved a saturating diversity after 150 bp. To assess the sensitivity of these diversity metrics to read length, Wilcoxon tests were performed for each diversity index to assess differences between disturbed and undisturbed samples (Figure S2). No consistent results were found. For richness, the W‐statistic significantly decreased or increased (datasets 2 and 3, respectively, *p* < 0.01); or exhibited no patterns, as was the case with 7 datasets. When assessed in terms of inverse Simpson's index, datasets 2 and 9 had greater discrimination between disturbed and undisturbed samples with longer read lengths (*p*‐value < 0.01). When assessed in terms of Faith's PD, only dataset 4 exhibited stronger discrimination with longer reads (*p*‐value < 0.001).

**FIGURE 4:**
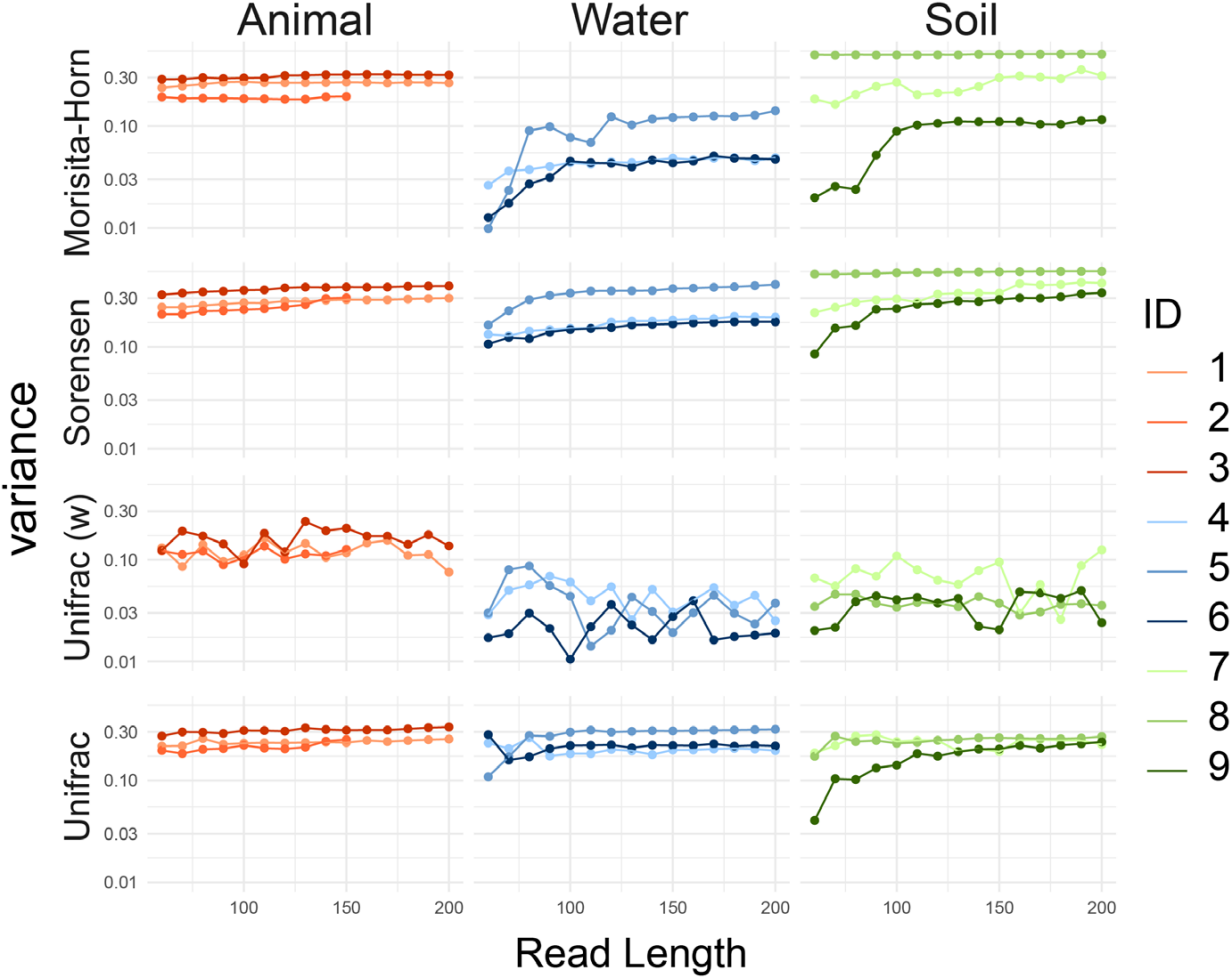
Variance in beta diversity across read lengths. For each dataset and for each read length, variance was calculated as the mean distance to the centroid among undisturbed samples using Morisita‐Horn (weighted) and Sørensen (unweighted) indices, as well as the weighted and unweighted Unifrac distances. Differences in variance between disturbed and undisturbed samples for each dataset are displayed in Figure S3.

The extent to which shorter read lengths affected the variance in compositional metrics (i.e., beta diversity) was assessed by measuring the mean distance to centroids among undisturbed samples using four dissimilarity metrics. With the exception of datasets 1 and 2, increasing read length resulted in increasing between‐sample variance when assessed in terms of Morisita‐Horn dissimilarities (Kendall's tau 0.63–0.81; *p*‐value < 0.001 for all comparisons). Sorensen dissimilarities exhibited stronger patterns (Kendall's tau 0.89–0.99; *p*‐value < 0.001 for all comparisons). No patterns were detected with weighted Unifrac distances, but datasets 1,2, 3, 5, and 9 exhibited increasing variance with longer reads when assessed with unweighted Unifrac distances (Figure 4).

**FIGURE 5:**
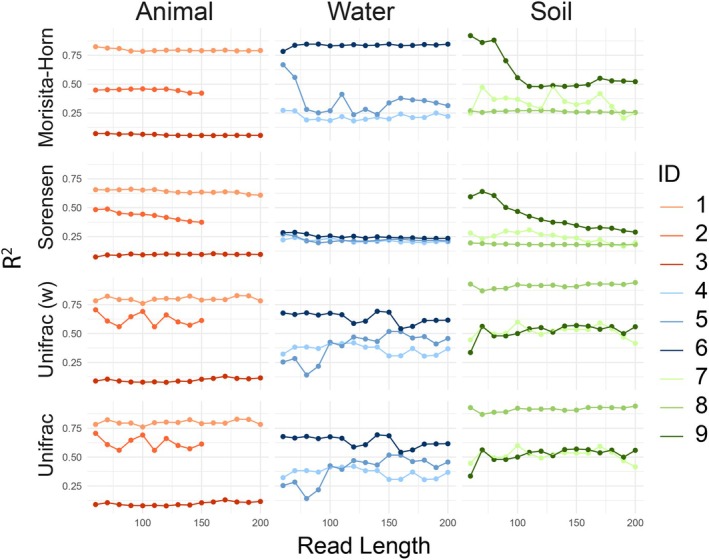
Discrimination between ecological states across read lengths. For each dataset, PERMANOVAs between the undisturbed and disturbed samples were assessed for Morisita‐Horn (weighted) and Sorensen (unweighted) indices, as well as the weighted and unweighted Unifrac distances assessed across read lengths. Each point represents the *R* ^2^ value for each test, and associated *p*‐values are available in Figure S4.

The extent to which shorter read lengths recovered differences in beta diversity between the undisturbed and disturbed samples was evaluated for each dataset (Figure 5) using PERMANOVAs. No consistent patterns in discrimination were observed with increasing read lengths. With Morisita–Horn dissimilarities, dataset 3 exhibited a decreasing *R* ^2^ and increasing *p*‐values (Figure S4) with longer reads (Kendall's tau −0.66, *p* < 0.001), while with Sorensen's dissimilarities, all datasets except for 3,4, and 5 exhibited a decreasing *R* ^2^ with longer reads (Kendall's tau −0.59 to −0.95; *p*‐value < 0.002 for all comparisons), but no trend in *p*‐values (Figure S4). No patterns were found with weighted or Unifrac distances (*p* > 0.01 for all comparisons).

**FIGURE 6:**
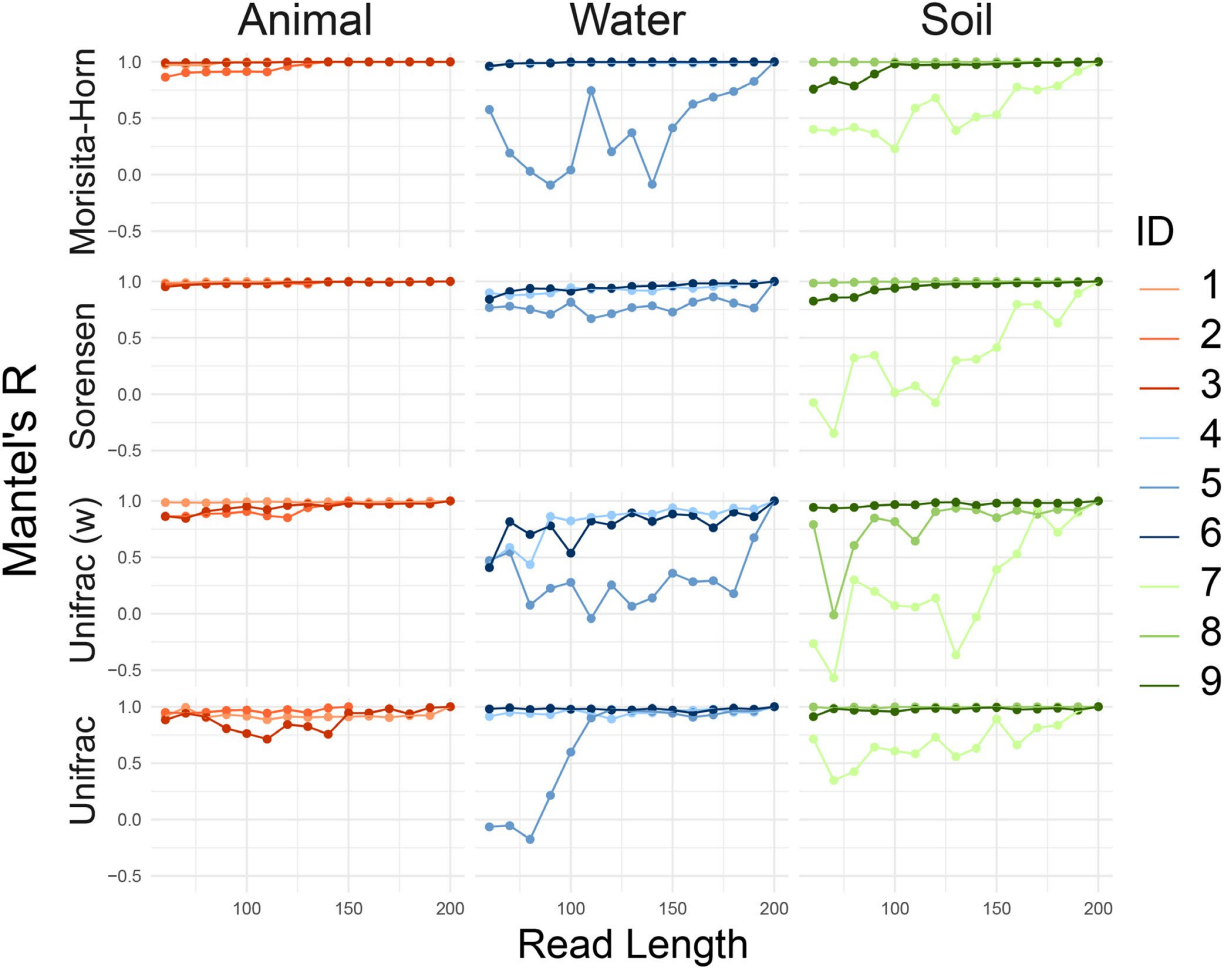
Information loss from shorter read lengths. For each study and each read length, Morisita‐Horn (weighted) and Sorensen (unweighted) indices, as well as the weighted and unweighted Unifrac distances were calculated for the 200‐bp reads and each shorter read length to evaluate the correlation between shorter read lengths and the most information‐rich version of the dataset (200 bpp).

Finally, to further examine information loss from shorter read lengths, Mantel tests between the communities resulting from each read length and the dataset trimmed to 200 bp (i.e., the most information‐rich version) were performed for each beta diversity metric (Figure 6, Figure S4). When evaluated with Morisita‐Horn dissimilarities, Mantel's statistic increased in all datasets except for 8 and 4 (Kendall's tau 0.54–0.89; *p*‐values < 0.005 for all comparisons). Similarly, significant increases in Mantel's statistic with read length were found for all datasets except for 1, 2, and 5 using both Sorensen's dissimilarities and Unifrac distances (Kendall's tau 0.54–0.98, *p*‐value < 0.005 or all comparisons). With weighted Unifrac distances, only datasets 5, 7, and 8 exhibited positive relationships between read length and Mantel's statistic (Kendall's tau 0.60–0.77; *p*‐values < 0.002 for all comparisons).

## 4 Discussion

Amplicon sequencing remains the most common method for characterising microbial communities, largely due to its low price and high throughput relative to long‐read amplicon sequencing and shotgun metagenomics. As this technique becomes increasingly accessible around the world, the 16S rRNA sequence archives are likely to continue growing. Understanding how processing choices affect the estimation of microbial diversity is essential for the interpretation and reuse of amplicon sequencing data (Jurburg et al. 2022). This work evaluated how shorter read lengths affect the number of observations, the number and taxonomic classification of ASVs, and the biodiversity estimates derived to establish a set of minimum best practices for reprocessing amplicon sequence data comparably—an essential step in data reanalysis. Its findings indicate that short read lengths recover biodiversity patterns, but special caution should be taken in the selection of biodiversity metrics to examine these data.

Across all datasets and environments, processing sequence data to shorter read lengths preserved more reads per sample, and this also resulted in the ability to preserve more reads during rarefaction, which remains a central component of ecological analyses of microbiome data (Schloss 2024). In the context of data reuse, resolution gained from increasing the length of a sequenced DNA segment has received much attention, but increasing resolution by increasing the number of observations considered has been generally ignored. This work demonstrates a direct trade‐off between both sources of ecological resolution that come at different costs: shorter read lengths resulted in a higher proportion of ASVs lacking classification at higher taxonomic resolutions (Wang et al. 2007) but a greater number of sequences or observations.

This study highlights the possibility for educated compromises in resolution: for all datasets, only marginal improvements in taxonomic assignments were obtained by read lengths greater than 150 bp at the family or coarser levels. This suggests that if only forward reads are available, little taxonomic information is lost by 150 bp reads relative to the full forward read. Importantly, classification was best in the animal datasets, which are the least diverse and best‐characterised systems. These results also highlight that genus‐level taxonomic assignments greatly depend on how well‐characterised the microbiota of the target environment are, and support previous recommendations that interpretations of genus‐level assignments are not recommended for shorter reads (Thompson et al. 2017); a large proportion of the community may be unclassified.

The detection of ASVs and the resulting alpha and beta diversity were more robust to read length than fine taxonomic classification, especially when abundance‐weighted metrics were employed (i.e., Inverse‐Simpson index, weighted Unifrac, and Morisita‐Horn dissimilarities), at shorter read lengths, exhibiting fewer trends. Reads as short as 90 bp could consistently recover the dissimilarity between communities belonging to both biological replicates (i.e., variance or dispersion) and different treatments; however, the recovery of alpha diversity additionally depended on the inherent diversity of the system. Notably, in general, methods that considered phylogenies (Faith's PD, weighted and unweighted Unifrac) were more robust to changes in read lengths, but as with the other metrics, weighted Unifrac was more robust than its unweighted counterpart. Importantly, the similarity between the 200 bp datasets and their shorter versions increased with read length when assessed with presence‐based Sorensen dissimilarities, but remained high for abundance‐weighted dissimilarities, even for the shortest reads. The differences observed when using weighted and unweighted metrics suggest that shortening read lengths disproportionately decreases the abundance and presence of rare ASVs, highlighting the dependence of rare ASVs on the length of the DNA segment used.

Similarly, richness estimates increased linearly with read length until a saturation point that aligned with the expected diversity in each environment explored (i.e., from least to most diverse, the animal, aquatic and soil microbiomes), emphasising the importance of defining diversity estimates relative to the sequence read length. These results highlight the importance of considering diversity estimates, particularly incidence‐based alpha diversity metrics (i.e., richness) within the context of the read length selected, rather than as absolute measures. In the case of data reuse and comparison among datasets, this study demonstrates the importance of applying a uniform read length across datasets in order to have comparable diversity estimates.

Using short read lengths, especially within a synthesis context, may increase the number of datasets that can be integrated and compared while increasing the number of observations used per sample. First, using only forward reads may increase the number of suitable datasets for re‐analysis while reducing cross‐study biases. A previous study estimated that at least 16.8% of the 16S rRNA gene amplicon sequence datasets currently stored in International Nucleotide Sequence Database Collaboration archives (e.g., NCBI's sequence read archives) are categorised as pair‐ended data but have only archived forward reads (Jurburg et al. 2020). In other cases, datasets may be archived in their merged format, resulting in additional uncertainty in the location of merging, where quality is often lower. Second, with Illumina‐derived data, sequence quality decreases with read length (Callahan, Sankaran, et al. 2016). Consequently, fewer reads pass quality checking, resulting in fewer reads (or observations) in the final, processed dataset. Third, different studies employ different sequencing platforms, which produce reads of variable lengths, the shortest of which is Illumina HiSeq, featuring a maximum read length of 150 bp, often including barcodes and/or primers (Di Bella et al. 2013).

This work demonstrates how one aspect of sequence processing (i.e., trimming) affects inferences on the taxonomic resolution, alpha, and beta diversity patterns of microbial metabarcoding data, as well as the conclusions that can be derived from them. While several studies have examined how technical choices (i.e., primer choice (Fouhy et al. 2016; Martínez‐Porchas et al. 2016; Tremblay et al. 2015), pipeline selection (Marizzoni et al. 2020), and rarefaction (McKnight et al. 2018; Schloss 2024; Weiss et al. 2017)) affect the estimation of diversity, systematic assessments of how other technical choices (particularly bioinformatics parameters e.g., chimera checking) affect the microbial diversity estimates are lacking but urgently needed (Calderón‐Sanou et al. 2020). Importantly, short reads enable the reuse of sequence data in their rawest form, allowing for complete and unified reprocessing of the sequence data from different studies, which may in turn improve comparability among them (Kang et al. 2021).

Processing metabarcoding data involves making a series of choices that affect the final dataset and its interpretation (Abellan‐Schneyder et al. 2021). Sequence trimming is a critical part of processing, but its effect on the resulting diversity estimates are often overlooked. The analyses presented focused on the effect of sequence trimming in the popular *dada2* pipeline, which detects amplicon sequence variants (ASVs) rather than grouping sequences into clusters of 97% sequence similarity. While the findings in this study may guide the general processing of amplicon sequencing data, it is important to note that the findings are specific to the *dada2* pipeline. From a data reanalysis perspective, the determination of ASVs is scalable, which is important for large datasets, and results in universal taxonomic units, which may facilitate comparisons across studies in the future. *dada2* has been extensively validated and exhibits high sensitivity to ASVs (Prodan et al. 2020). Multiple steps in the *dada2* pipeline are affected by varying read lengths. For example, the increase in the number of reads following processing results from discarding the ends of the reads, which have a lower average quality, and therefore do not pass the quality‐based filtering parameters. *dada2* also uniquely discards singleton ASVs, which are likely more abundant for longer read lengths, but also in more diverse environments. Indeed, the consistent decrease in *R* ^2^ values for beta diversity analyses across longer reads likely reflects an increase in the complexity of the data, which in turn reflects the extreme complexity of bacterial communities.

This study also represents the ‘best case scenario’ of data reuse, as the datasets were selected to use the most common primer region, and the most common platform (Illumina; (Jurburg et al. 2020)). Relative to other fields (e.g., medicine), data reuse in microbiome research is still in its infancy, but sequence data are abundant and available. Further work should focus on the integration of datasets targeting different gene regions (Wasimuddin et al. 2020) and explore the potential for merging sequence data from different sequencing platforms, which, while common in other areas of bioinformatics (i.e., shotgun metagenomics), is uncommon in metabarcoding data.

## 5 Conclusion

This study lays the groundwork for the analysis and reanalysis of partial metabarcoding data using short read lengths, and results in several recommendations. First, when comparing data with different technical backgrounds (i.e., from different studies), trimming to the same read length is important, especially for the analysis of alpha diversity, as alpha diversity is directly affected by read length. Second, when using short read lengths, caution should be taken with the interpretation of genus‐level classifications. Third, abundance‐weighted diversity metrics (i.e., inverse Simpson index, weighted Unifrac, Morisita‐Horn dissimilarity) are more robust to read length than incidence‐based metrics (i.e., richness and Sorensen dissimilarity). Finally, the estimation of microbial diversity from sequence data is far from absolute and should instead be considered relative to the read length employed.

## Author Contributions

S.D.J. designed the study, selected the data, performed the analyses and wrote the manuscript.

## Conflicts of Interest

The author declares no conflicts of interest.

## Supporting information


**Data S1.** Supplementary Figures.

**Data S2.** Supplementary Tables.

## Data Availability

Accession numbers for each of the nine datasets reused as well as the DOIs of their associated publications are available in Table 1. Data for this study was downloaded from NCBI's Sequence Read Archives. Code and metadata used to generate the analyses presented is available at https://github.com/drcarrot/ReadLength.
