# Supplementary material for: Short Read Lengths Recover Ecological Patterns in 16S rRNA Gene Amplicon Data
Source: Mol Ecol Resour. 2025 Mar 13;25(6):e14102. doi: 10.1111/1755-0998.14102 (PMC12225704; doi:10.1111/1755-0998.14102)
Supplement: Supplementary file 1 — Data S1. Supplementary Figures. [file MEN-25-e14102-s001.pdf]

MOLECULAR ECOLOGY RESOURCES

Supplemental Information for:

Short read lengths recover ecological patterns in 16S rRNA gene amplicon data

Stephanie Jurburg

Supplementary materials and methods

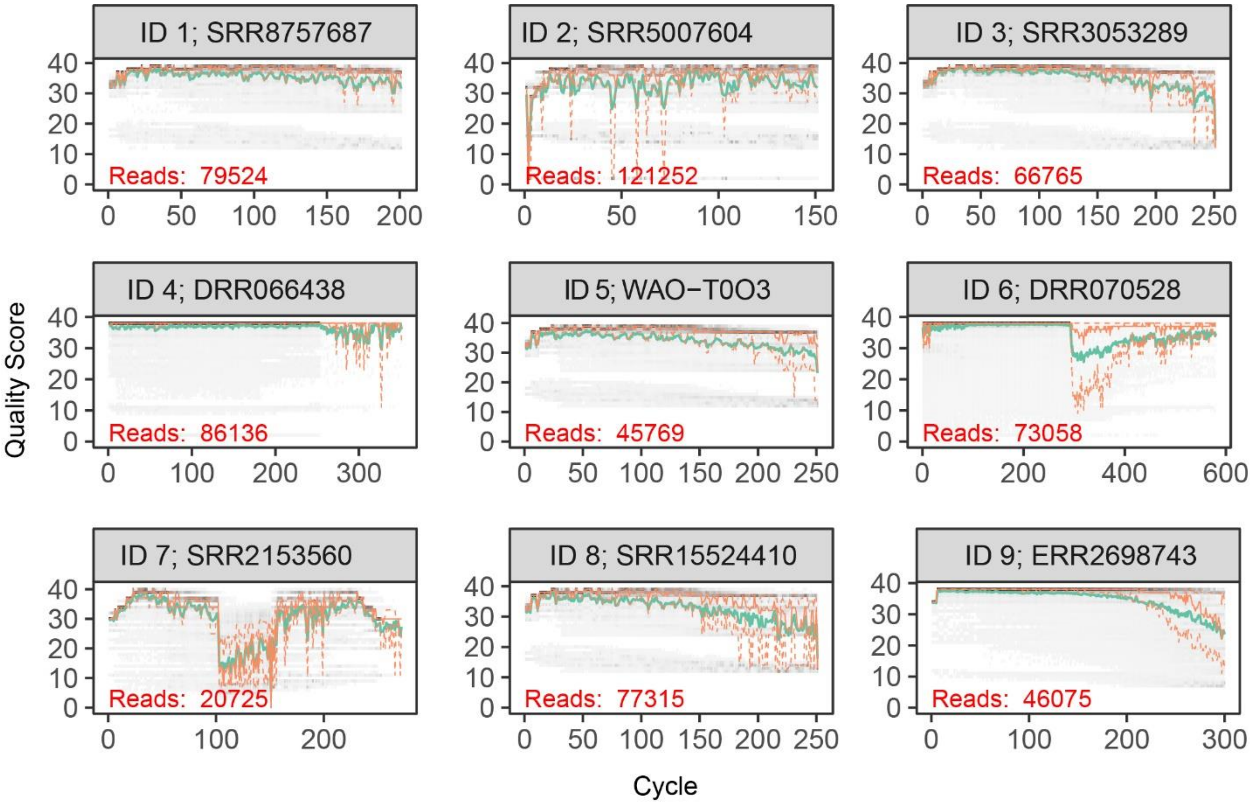

Figure S1. Quality profiles for selected samples in each study. Each panel shows the quality profile for a randomly selected undisturbed sample in one dataset. The gray scale indicates the frequency of each quality score at each base position (darkness indicates a higher frequency). Green and orange lines indicate the mean and quartile quality score at each position, respectively.

MOLECULAR ECOLOGY RESOURCES

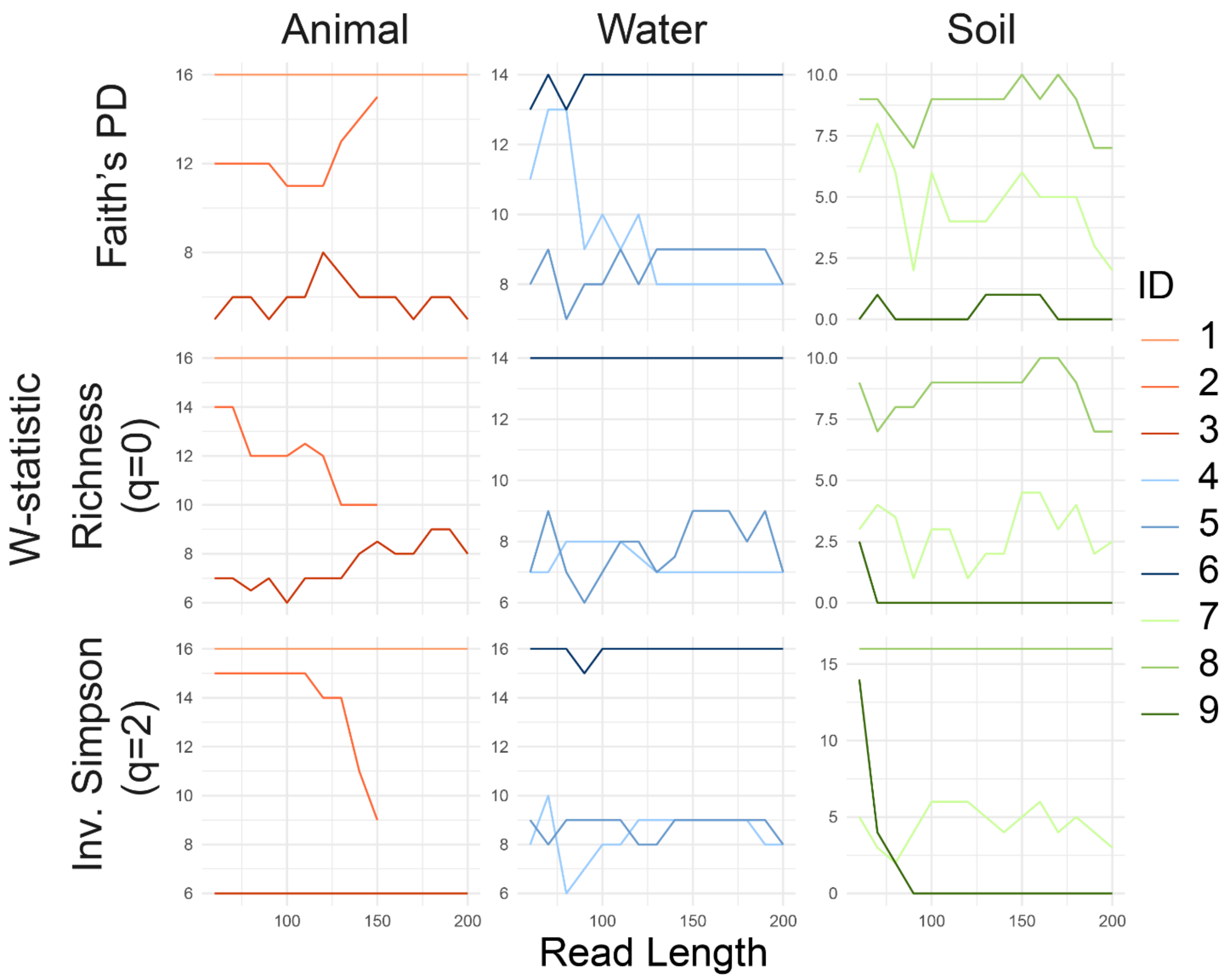

Figure S2. W-statistic for a Wilcoxon test of the alpha diversities (Faith's phylogenetic diversity, richness, and inverse Simpson's index) estimated in the disturbed and undisturbed samples in each dataset, for each read length.

MOLECULAR ECOLOGY RESOURCES

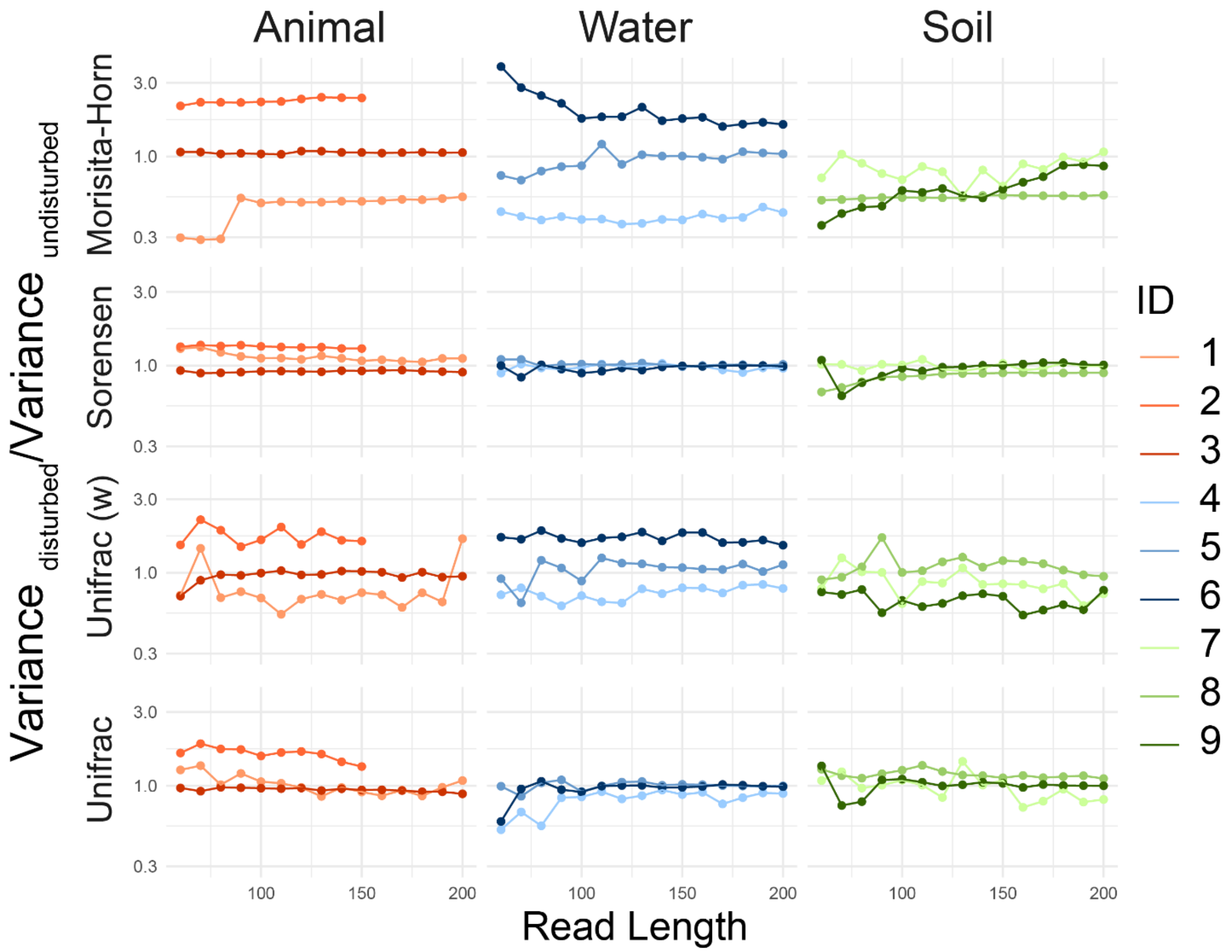

Figure S3. Differences in variance in beta diversity between disturbed and undisturbed samples for each dataset, for each read length, evaluated as the ratio of variance in the disturbed to the undisturbed samples.

MOLECULAR ECOLOGY RESOURCES

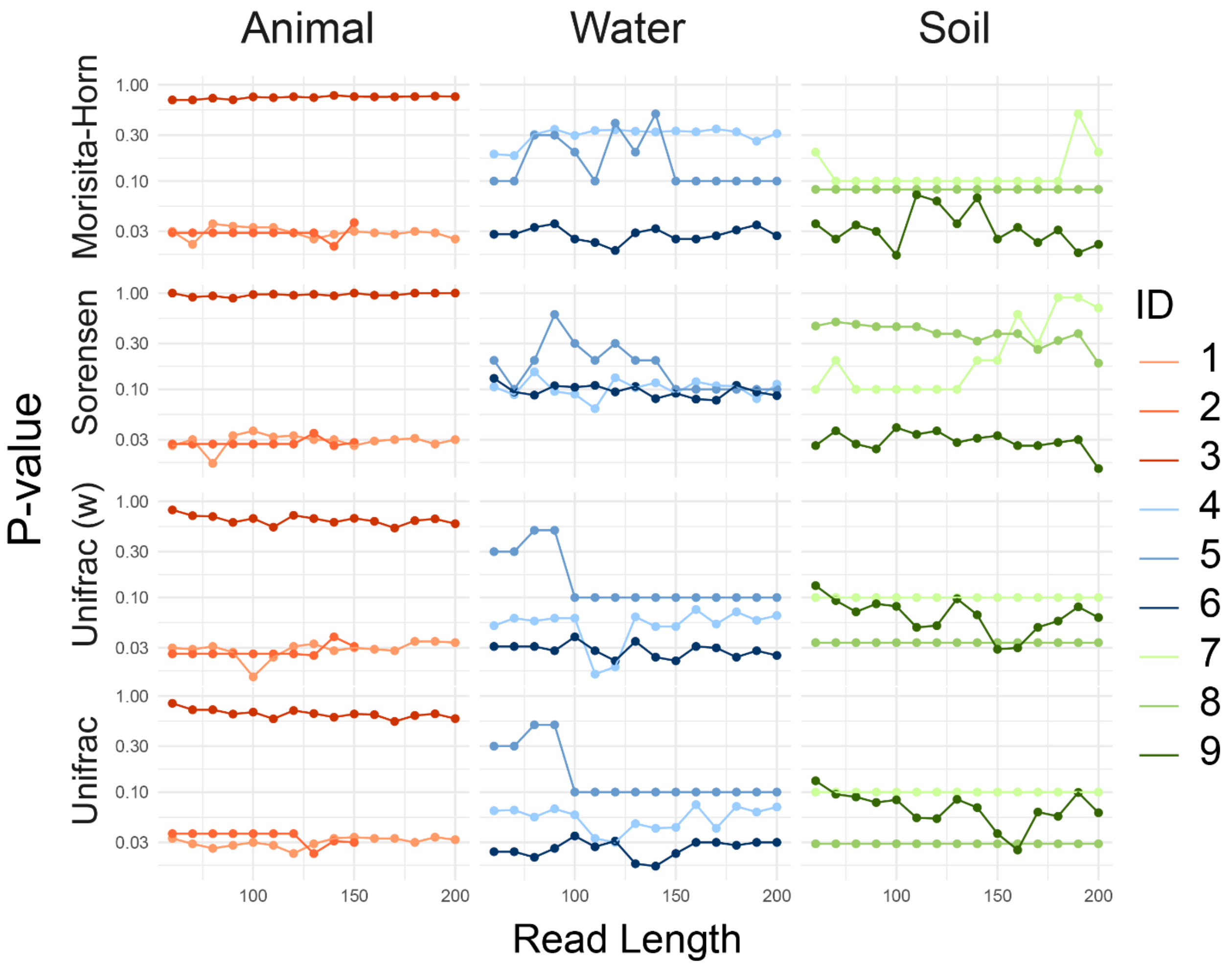

Figure S4. P-values for the PERMANOVA tests performed. . For each dataset, PERMANOVAs between the undisturbed and disturbed samples were assessed for Morisita-Horn (weighted) and Sorensen (unweighted) indices, as well as the weighted and unweighted Unifrac distances were assessed across read lengths. Each point represents the R2 value for each test
